# Experimental Investigation of the Impact of Niobium Additions on the Structural Characteristics and Properties of Ti–5Cr–xNb Alloys for Biomedical Applications

**DOI:** 10.3390/ma17071667

**Published:** 2024-04-04

**Authors:** Hsueh-Chuan Hsu, Shih-Ching Wu, Wei-Ching Fang, Wen-Fu Ho

**Affiliations:** 1 Department of Dental Technology and Materials Science, Central Taiwan University of Science and Technology, Taichung 406053, Taiwan; hchsu@ctust.edu.tw (H.-C.H.); scwu@ctust.edu.tw (S.-C.W.); 2 Department of Materials Science and Engineering, Da-Yeh University, Changhua 515006, Taiwan; 3 Department of Chemical and Materials Engineering, National University of Kaohsiung, Kaohsiung 811726, Taiwan

**Keywords:** titanium alloys, microstructure, microhardness, mechanical properties

## Abstract

In this study, a series of Ti–5Cr–xNb alloys with varying Nb content (ranging from 1 to 40 wt.%) were investigated to assess their suitability as implant materials. Comprehensive analyses were conducted, including phase analysis, microscopy examination, mechanical testing, and corrosion resistance evaluation. The results revealed significant structural alterations attributed to Nb addition, notably suppressing the formation of the ω phase and transitioning from α′ + β + ω to single β phase structures. Moreover, the incorporation of Nb markedly improved the alloys’ plastic deformation ability and reduced their elastic modulus. In particular, the Ti–5Cr–25Nb alloy demonstrated high values in corrosion potential and polarization resistance, signifying exceptional corrosion resistance. This alloy also displayed high bending strength (approximately 1500 MPa), a low elastic modulus (approximately 80 GPa), and outstanding elastic recovery and plastic deformation capabilities. These aggregate outcomes indicate the promising potential of the β-phase Ti–5Cr–25Nb alloy for applications in orthopedic and dental implants.

## 1. Introduction

Titanium (Ti) and its alloys are highly esteemed metallic biomaterials due to their remarkable mechanical properties, superior biocompatibility, and robust corrosion resistance. They are extensively employed in orthopedic and dental applications [1]. Ti–6Al–4V, a widely utilized implant material, has a significantly higher elastic modulus than bone, potentially leading to stress shielding and resultant bone osteoporosis [2]. Reducing the elastic modulus is thus pivotal for enhancing the biomechanical compatibility of Ti alloys.

Concerns have been raised regarding the leaching of aluminum, vanadium, and nickel ions from biometals, such as Ti–6Al–4V and Ti–Ni (nitinol) alloys, due to potential long-term health issues [3,4]. These ions have been associated with neurological disorders, enzymatic disturbances, and toxicity, and even carcinogenicity in the case of nickel. Consequently, there is growing interest in the exploration of novel Ti-based alloys incorporating biocompatible alloying elements.

Various binary Ti alloys, such as Ti–Cr [5], Ti–Nb [6], Ti–Mo [6], and Ti–Sn [7], have been systematically evaluated for dental and orthopedic applications. However, these binary alloys still require further refinement of their mechanical properties, particularly in terms of reducing the elastic modulus. Additionally, Niinomi [8] reported that the elastic moduli of β-type Ti alloys were lower than those of α- or α + β-type Ti alloys. Consequently, there has been growing interest in low-modulus β-type titanium alloys incorporating toxic-free alloying elements. Ti alloys with a low elastic modulus and incorporating Nb as a β-phase stabilizer in biomedical applications have been explored [9,10]. Moreover, the excellent biocompatibility of titanium alloys is a crucial factor for their use as biomedical implants. Common β-stabilizing elements possess outstanding corrosion resistance, such as Nb, Zr, Ta, and Mo [11,12,13,14]. Incorporating these elements into titanium alloys can enhance the stability of the passivation layer, preventing excessive release of metal ions from biomedical implants during clinical applications.

In our prior investigation [5], the as-cast Ti–5Cr alloy demonstrated higher bending strength compared to CP-Ti and the lowest bending modulus among all binary Ti-Cr alloys. Unfortunately, Ti–5Cr exhibited reduced bending deflection due to ω phase-induced embrittlement. In the current study, the impact of varying amounts of Nb element on the structures and properties of Ti–5Cr-based alloys was investigated, aiming to assess the suitability of these novel alloys for biomedical implant applications.

## 2. Materials and Methods

### 2.1. Alloy Fabrication

Ti–5Cr–xNb alloys (x = 0, 1, 5, 10, 15, 20, 25, 30, 35, and 40 wt.%) were fabricated using Ti (99.7% purity), Cr (99.95% purity), and Nb (99.95% purity) in a commercial arc-melting vacuum-pressure-type casting system. Commercial pure Ti (CP-Ti, 99.7% purity) served as a reference material. To ensure compositional homogeneity, each ingot, weighing approximately 15 g, underwent five cycles of flipping and remelting. Throughout the arc-melting procedure, a preheated Ti getter was employed prior to melting the reactant mixture to enhance the purification of the argon atmosphere. Subsequently, the ingots underwent an additional melting stage before casting, following a previously described casting procedure [15].

### 2.2. Microstructural and Phase Analysis

For microstructural analysis, the specimens underwent grinding and polishing according to established metallographic protocols, culminating in a final polishing step utilizing 0.3 μm alumina powder. Following this, the specimens were etched in a solution comprising 5 vol.% HF, 15 vol.% HNO_3_, and 80 vol.% H_2_O. The microstructure of the etched alloys was examined using an optical microscope (OM; BH2, Olympus, Tokyo, Japan). Phase identification was performed by X-ray diffraction (XRD) using a diffractometer (XRD-6000, Shimadzu, Kyoto, Japan) operating at 30 kV and 30 mA. Ni-filtered Cu Kα radiation was utilized, and phase identification involved matching characteristic peaks with the JCPDS files. XRD patterns were recorded in the 30–80° (2θ) range, with a step size of 0.02° and a scan rate of 4°/min. A lower scanning rate of 0.5°/min was employed to detect the ω phase over a 2θ range of 74–86°.

### 2.3. Mechanical Testing

**Figure 1:**
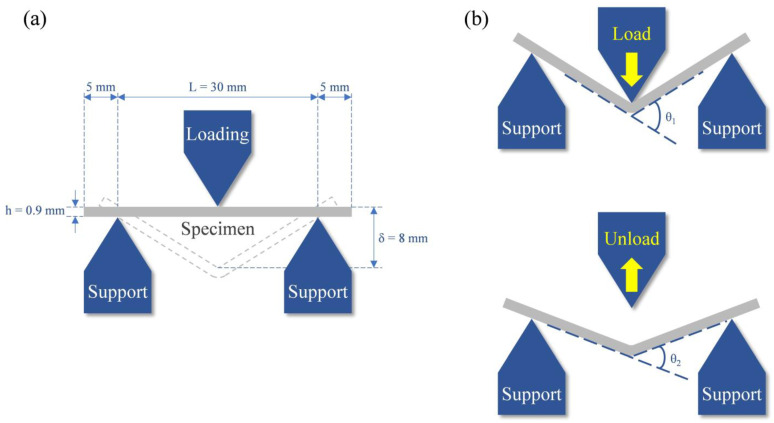
Schematic diagram of (**a**) three-point bending test and (**b**) elastic recovery angles measurement.

Microhardness assessments of the polished alloys were performed employing a micro-hardness tester (MVK-E3, Mitutoyo, Kawasaki, Japan), applying a 100 g load for 15 s. Three specimens were tested for each alloy, and five measurements were taken at different positions on each specimen. The reported values represent the averages of these measurements. Bending strength was assessed via a three-point bending test conducted on a desktop mechanical tester (AG-IS, Shimadzu, Kyoto, Japan) at a crosshead speed of 0.5 mm/min. The schematic diagram of the three-point bending test (Figure 1a) and measurement of elastic recovery angles (Figure 1b) are illustrated in Figure 1. Bending strength (σ) was calculated using the equation $\sigma =\frac{3PL}{2bh^{2}}$ [16], where P is the load (N), L is the span length (mm), b is the specimen width (mm), and h is the specimen thickness (mm). Additionally, the elastic modulus (E) was determined using the equation $E=\frac{PL^{3}}{4bh^{3}∆\delta }$ [16], where Δδ represents the load displacement. Elastic recovery angles were measured as θ_1_ minus θ_2_. Prior to the bending test, the entire surface layer of each specimen was ground using SiC abrasive paper, resulting in specimens with dimensions of length approximately 30 mm, width around 5 mm, and thickness about 0.9 mm. The average bending strength and elastic modulus were calculated for at least five specimens of each alloy type. For specimens that fractured during the bending test, the fracture surfaces were analyzed using a scanning electron microscope (SEM; JSM-6700F, JEOL, Tokyo, Japan).

### 2.4. Corrosion Testing

**Figure 2:**
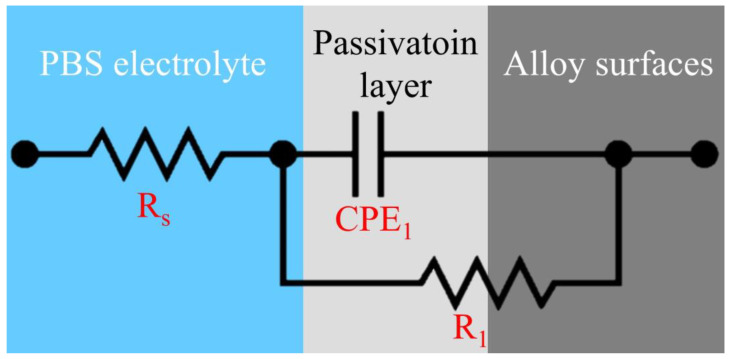
The equivalent electrical circuit model utilized for fitting EIS data.

The corrosion behavior of the Ti–5Cr–xNb specimens was assessed utilizing a Potentiostat (PGSTAT12, Autolab, Utrecht, The Netherlands) in phosphate-buffered saline (PBS) at 37 °C and pH 7.4. The Ti–5Cr–xNb specimen served as the working electrode, a silver chloride electrode (Ag/AgCl) as the reference electrode, and a platinum plate as the auxiliary electrode. For potentiodynamic polarization tests, the open circuit potential (OCP) of each specimen was monitored for 60 min, with a scan rate of 1 mV/s and scan ranges set at −0.6 to 1.6 V. Electrochemical impedance spectroscopy (EIS) was employed to analyze the passivation layer properties of the alloy. This involved a voltage amplitude of 10 mV and a frequency range spanning from 10^5^ to 10^−^^2^ Hz. Figure 2 presents the equivalent electrical circuit (EEC) model utilized for fitting EIS data. In this EEC model, R_s_ represents the electrolyte resistance, CPE_1_ signifies the constant phase element of the passivation layer, and R_1_ represents the polarization resistance of the passivation layer. The experimental data were fitted and analyzed using the Metrohm Autolab NOVA 2.1 software. The PBS solution used in these experiments consisted of NaCl (8.0 g/L), KCl (0.2 g/L), Na_2_HPO_4_ (1.44 g/L), and KH_2_PO_4_ (0.24 g/L).

## 3. Results and Discussion

### 3.1. Phase Identification

**Figure 3:**
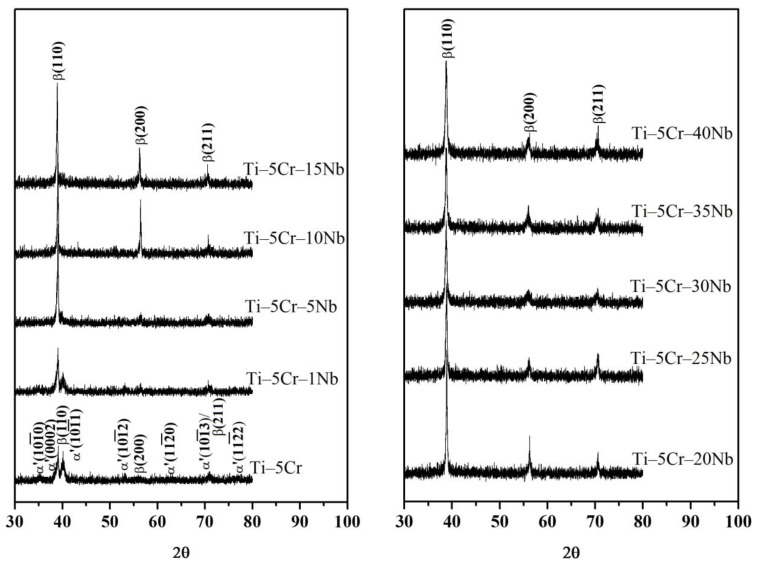
X-ray diffraction patterns of Ti–5Cr and Ti–5Cr–xNb alloys acquired in the 30–80° (2θ) range, utilizing a step size of 0.02° and a scan rate of 4°/min.

The XRD patterns of Ti–5Cr and various Ti–5Cr–xNb alloys are illustrated in Figure 3. The crystal structure of Ti–5Cr–xNb alloys is significantly influenced by the Nb content. As depicted in Figure 3, Ti–5Cr and Ti–5Cr–1Nb alloys predominantly display the α′ + β phase. However, with an increase in Nb content, the intensities of the α′ phase decrease. Beyond a Nb content of 5 wt.% in the Ti–5Cr–xNb alloy, the α′ phase is effectively suppressed, leaving the exclusive presence of a metastable β phase.

**Figure 4:**
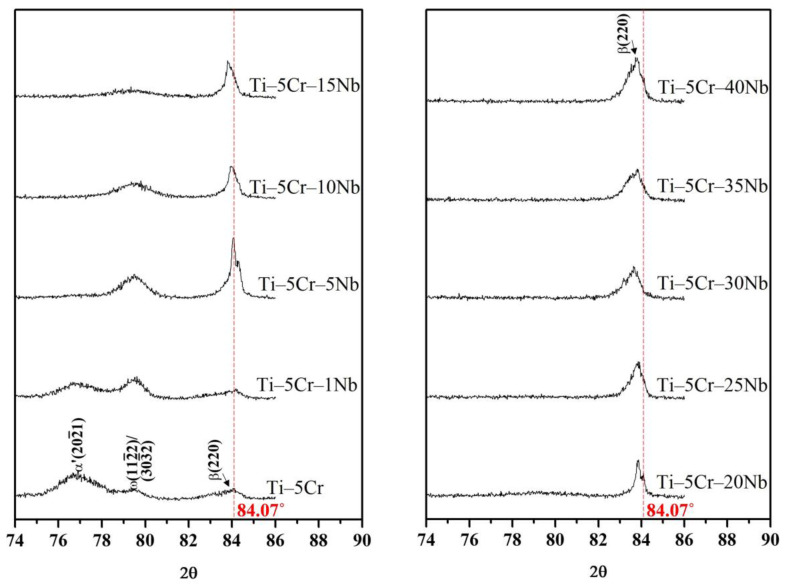
X-ray diffraction patterns of Ti–5Cr and Ti–5Cr–xNb alloys obtained in the 74–86° (2θ) range, employing a step size of 0.02° and a scan rate of 0.5°/min.

In the Ti–5Cr specimen, only peaks corresponding to the α′ + β phase were detected, and no ω phase peaks were evident in Figure 3. In a previous investigation [5], the presence of the ω phase was successfully identified using XRD analysis at a lower scanning speed (0.5°/min). As depicted in Figure 4, the ω phase was detected in both Ti–5Cr and the Ti–5Cr–xNb alloys (x = 1, 5, 10, and 15), although its presence was less pronounced in Ti–5Cr–15Nb. With increasing Nb content, the ω reflections in the Ti–5Cr–xNb (x = 20, 25, 30, 35, and 40) alloys disappeared. The amount of athermal ω phase in β-phase alloys is contingent upon the stability of the β phase [17]. Consequently, the emergence of the athermal ω phase is hindered in alloys with high β-phase stability. In this study, the quantity of athermal ω phase in Ti–5Cr–15Nb is lower than that in Ti–5Cr–10Nb, and the athermal ω phase was not reliably observed in β-phase Ti–5Cr–xNb alloys with a high Nb content. As discussed later in this paper, it is imperative to minimize the formation of the brittle ω phase; even in small quantities, it significantly impacts the mechanical properties of the alloy. The brittle ω phase exhibits the highest modulus and hardness among all phases present in Ti alloys [18]. In contrast, β-phase Ti alloys typically demonstrate excellent fatigue resistance characteristics [19,20,21].

Additionally, in Figure 4, the angle of the β(220) diffraction peak for Ti–Cr is indicated by a red dashed line at 84.07°. However, as the Nb content in the alloy increases, the stability of the β phase is enhanced, leading to an observed shift of the β(220) diffraction peak towards lower angles. Such peak shifts typically occur in multicomponent alloys where the presence of solute elements with different atomic radii distorts the lattice, thereby altering the lattice parameters [22]. Since the atomic radius of Nb (146 pm) is significantly higher than that of Cr (128 pm), the lattice distortion induced by the addition of Nb to Ti–Cr alloys may be the primary factor causing the peak shift of the β(220).

### 3.2. Microstructure

**Figure 5:**
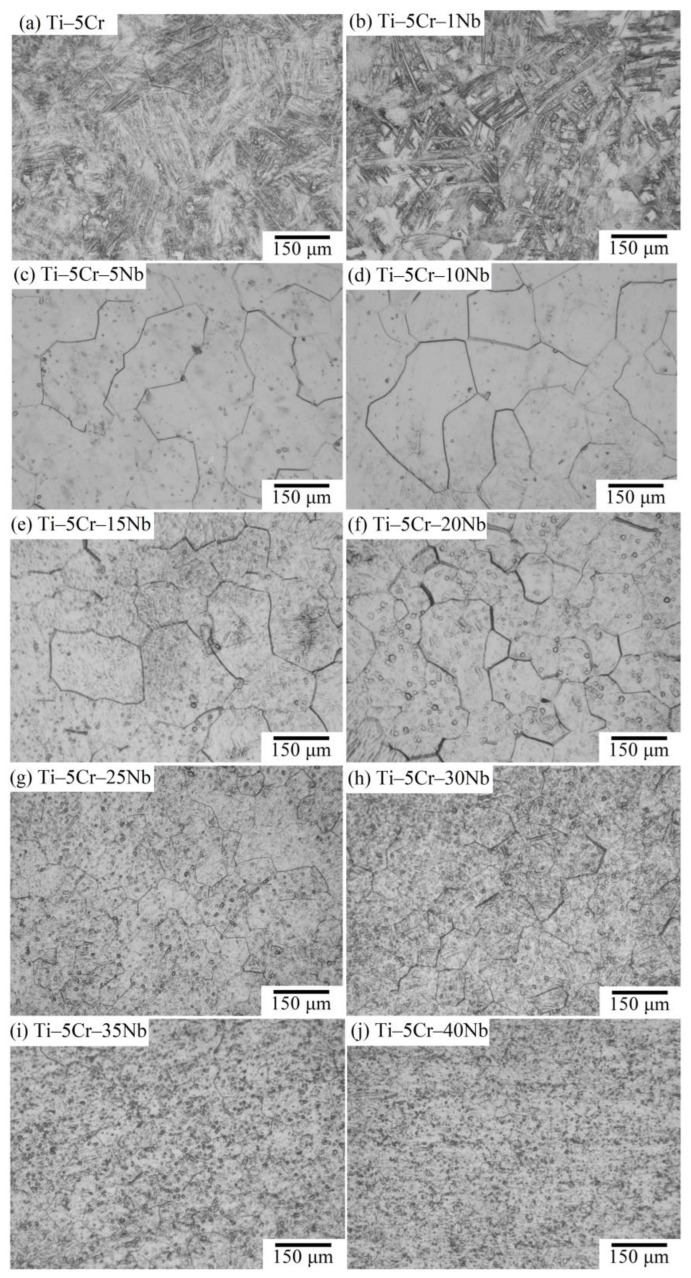
Optical micrographs showcasing the microstructures of Ti–5Cr and Ti–5Cr–xNb alloys.

Optical micrographs depicting the microstructures of cast Ti–5Cr and the series of Ti–5Cr–xNb alloys are presented in Figure 5. In Figure 5a, it is evident that the Ti–5Cr alloy displayed a hexagonal α′ phase, manifesting as a fine acicular martensite structure, while large original β grains were identifiable with distinct straight grain boundaries. This suggests that a substantial portion of the β phase undergoes transformation into the α′ martensite phase. Upon the addition of 1 wt.% Nb (Figure 5b), the microstructure still exhibited significant amounts of fine martensitic laths, but a well-retained lump of β phase was also discernible. For alloys containing 5 wt.% or more Nb (Figure 5c–j), all metallographic images showcased an equiaxial microstructure, indicative of retained β-type Ti alloys. In the Ti–5Cr–xNb alloy system, it becomes apparent that the β phase can be completely preserved during rapid cooling when the Nb content exceeds approximately 5 wt.%. The observed reduction in β phase grain size with increasing Nb content can be attributed to solid solution strengthening, which hinders dislocation movement and grain boundary diffusion, ultimately impeding grain growth. Additionally, lattice distortion caused by the increased Nb content also impedes grain growth [22]. This phenomenon aligns with findings in Ti–5Nb–xCr alloy systems [23]. In contrast to β-phase Ti–5Cr–xNb alloys without the ω phase, the Ti–5Cr–5Nb, Ti–5Cr–10Nb, and Ti–5Cr–15Nb alloys containing the ω phase exhibited significantly larger β grain sizes. This suggests that the presence of the ω phase did not exert as strong a constraint on the grain growth of the β phase. It is notable that alloys with smaller grain sizes often demonstrate higher strength and fatigue resistance [24].

### 3.3. Mechanical Properties

#### 3.3.1. Microhardness

**Figure 6:**
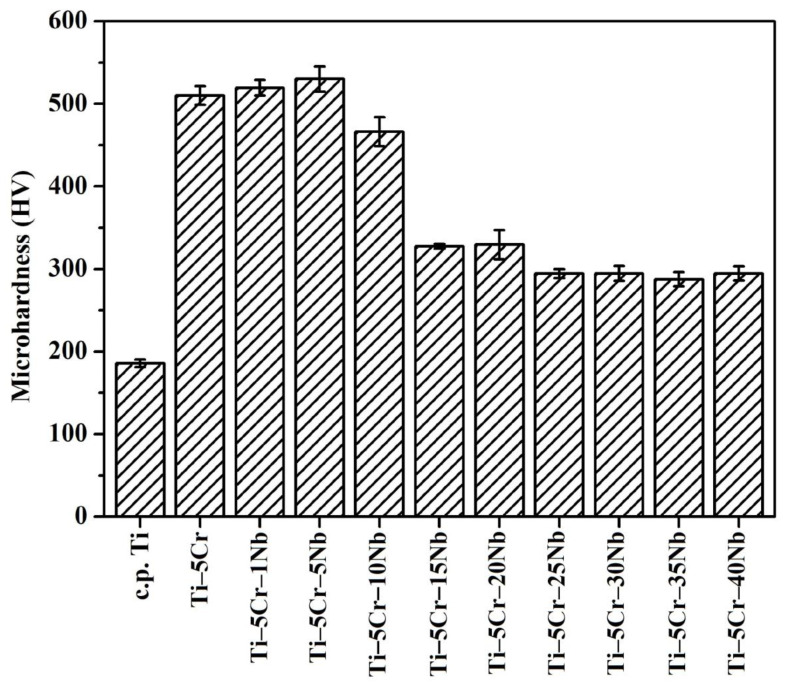
Microhardness variations in Ti–5Cr and Ti–5Cr–xNb alloys.

The microhardness values of as-cast CP-Ti, Ti–5Cr, and the Ti–5Cr–xNb alloy series are illustrated in Figure 6. Ti–5Cr and all Ti–5Cr–xNb alloys (ranging from 287 to 530 HV) exhibit significantly higher values compared to CP-Ti (186 HV). The increased microhardness in Ti–5Cr-based alloys can be attributed to the influence of the Cr and Nb elements, contributing either through solid solution strengthening or the effects of crystal structure/phases (α′, β, and ω). Except for Ti–5Cr–15Nb, characterized by a relatively small amount of ω phase, all Ti–5Cr–xNb (x = 0, 1, 5, and 10) alloys with a higher ω phase content demonstrate significantly elevated microhardness values compared to other Ti–5Cr-based alloys. Notably, Ti–5Cr–5Nb exhibits the highest microhardness (530 HV), surpassing that of Co–Cr–Mo (approximately 350 HV) [25] and Ti–6Al–4V (approximately 340 HV) [26], both widely used in biomedical applications.

#### 3.3.2. Bending Properties

**Figure 7:**
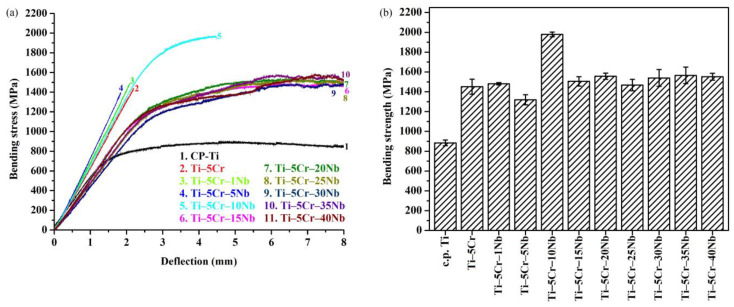
The bending properties of CP-Ti, Ti–5Cr, and Ti–5Cr–xNb alloys. (**a**) Bending stress-deflection profiles and (**b**) bending strengths.

The bending stress-deflection profiles of the alloy series and CP-Ti are depicted in Figure 7a. While Ti–5Cr and Ti–5Cr–xNb (x = 1, 5, and 10) alloys featuring the ω phase demonstrated brittleness and fracture, Ti–5Cr–15Nb, with a comparatively restricted ω phase, and other Ti–5Cr–xNb alloys devoid of the ω phase withstood the bending test without experiencing failure, even when subjected to the predetermined maximum deflection of 8 mm.

The bending strengths of CP-Ti, Ti–5Cr, and the Ti–5Cr–xNb alloys are presented in Figure 7b. Ti–5Cr–xNb alloys demonstrated significantly higher bending strengths (ranging from 1320 to 1979 MPa) than CP-Ti (852 MPa). Ti–5Cr–10Nb exhibited significantly higher bending strength than other Ti–5Cr-based alloys, approximately 2.3 times greater than that of CP-Ti. The enhanced strengths are likely attributed to either the solid-solution strengthening effect of the Cr and Nb alloying elements [27] or the hardening effect induced by the presence of the ω phase [28]. It is noteworthy that, compared to Ti–5Cr–10Nb, Ti–5Cr, Ti–5Cr–1Nb, and Ti–5Cr–5Nb exhibited excessively high ω phase contents in their phase structures. Consequently, these alloys experienced premature brittle fracture and demonstrated lower bending strength in their bending tests. In contrast, Ti–5Cr–15Nb showed only trace amounts of the ω phase in its phase structure, resulting in a bending strength similar to other single β phase Ti–5Cr–xNb alloys.

**Figure 8:**
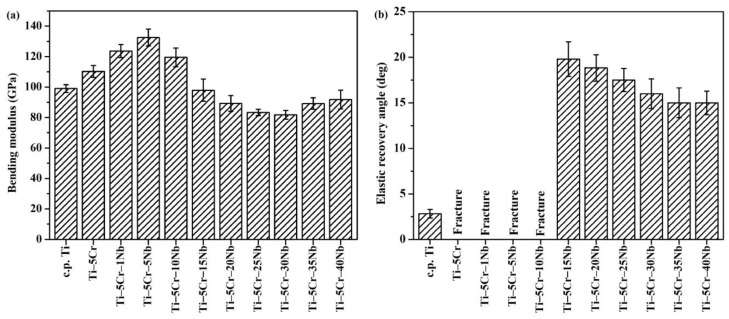
The elastic properties of CP-Ti, Ti–5Cr, and Ti–5Cr–xNb alloys. (**a**) Bending moduli and (**b**) elastic recovery angles.

Low elastic modulus is crucial for biomedical materials to reduce the stress-shielding effect and prevent bone resorption. The experimental results on elastic modulus are depicted in Figure 8a. Ti–5Cr–5Nb (133 GPa) exhibited significantly higher modulus than CP-Ti (98 GPa), Ti–5Cr (110 GPa), and other β-phase Ti alloys (ranging from 82 to 92 GPa). This observation could be linked to the occurrence of the ω phase formation during the quenching process. It is worth noting that Ti–5Cr–25Nb and Ti–5Cr–30Nb had a relatively lower bending modulus, 15% and 16% lower than CP-Ti, respectively.

In this study, all Ti–5Cr-based alloys, except for Ti–5Cr and Ti–5Cr–xNb (x = 1, 5, and 10) alloys with a significant amount of ω phase, exhibited excellent ductile properties. β-phase Ti–5Cr–xNb alloys without ω phase not only exhibited superior mechanical properties but also demonstrated notable elastic recovery capability. As depicted in Figure 8b, they exhibited significantly higher elastic recovery angles ranging between 15° and 20°, surpassing that of CP-Ti (2.7°).

In contrast to Ti–5Cr–30Nb, which demonstrates similar bending strength and elastic modulus, Ti–5Cr–25Nb exhibits a higher elastic recovery angle, thereby enhancing its operational lifespan in clinical applications. Furthermore, the cost of Ti–5Cr–25Nb is lower than that of Ti–5Cr–30Nb, attributed to the high cost of the Nb element. Therefore, Ti–5Cr–25Nb stands out as a competitively advantageous choice for use as an implant material. Hence, this study selects Ti–5Cr–25Nb for subsequent corrosion properties analysis.

#### 3.3.3. Fracture Surface Topography

**Figure 9:**
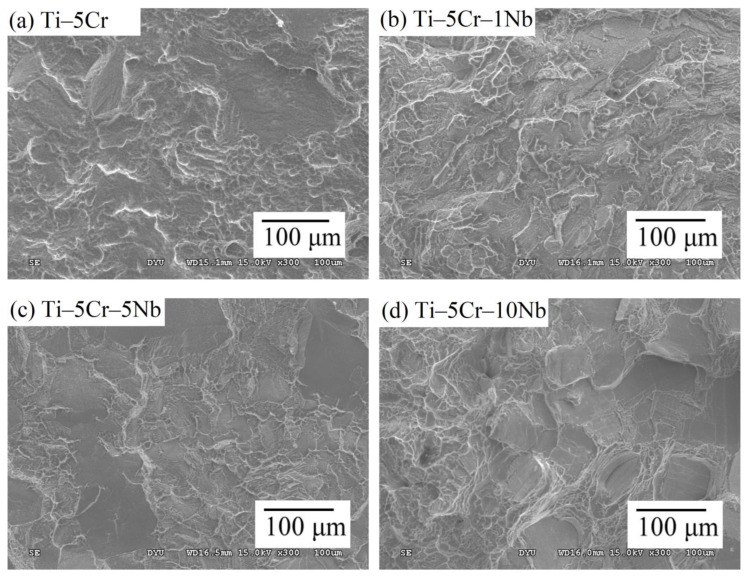
SEM fractography of Ti–5Cr–xNb alloys. (**a**) Ti–5Cr, (**b**) Ti–5Cr–1Nb, (**c**) Ti–5Cr–5Nb, and (**d**) Ti–5Cr–10Nb.

The impact of the ω phase is also evident in the fractography analysis of the current alloys. SEM examination of the fractured surfaces of bending specimens revealed distinct characteristics for Ti–5Cr, Ti–5Cr–1Nb, Ti–5Cr–5Nb, and Ti–5Cr–10Nb after completing the bending tests, as illustrated in Figure 9. Ti–5Cr and Ti–5Cr–1Nb exhibited similar fracture morphologies, marked by cleavage facets on the fractured surface, indicating reduced ductility. Some areas on the surface revealed numerous dimple ruptures, as depicted in Figure 9a,b. The fractured structure of Ti–5Cr–5Nb predominantly displayed cleaved facets with a few shallower dimples, as seen in Figure 9c. The dimples in this alloy were not as pronounced as those in the aforementioned alloys, aligning with the highly brittle failure observed, as indicated by a lower bending deflection value (approximately 1.8 mm). Contrarily, Ti–5Cr–10Nb showcased a fractured structure with numerous deep dimple ruptures and some cleavage facets. Deeper dimples in Ti–5Cr–10Nb, indicating greater ductility, were consistent with a fracture deflection of 3.8 mm, confirming its ductile fracture behavior.

### 3.4. Corrosion Properties

#### 3.4.1. Potentiodynamic Polarization Tests

**Figure 10:**
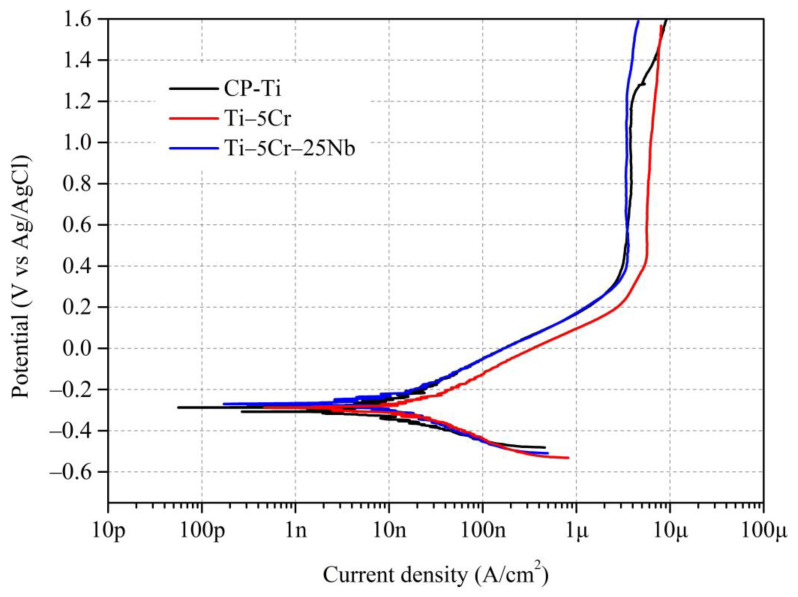
The polarization curves of CP-Ti, Ti–5Cr, and Ti–5Cr–25Nb in a PBS solution at 37 °C.

Biomedical materials need to exhibit excellent corrosion resistance to prevent corrosion or excessive ion release within the human body, which could adversely affect the patient’s health. In this study, three specimens, CP-Ti, Ti–5Cr, and Ti–5Cr–25Nb, were selected for subsequent corrosion resistance testing. Figure 10 presents the polarization curves of CP-Ti, Ti–5Cr, and Ti–5Cr–25Nb in a PBS solution at 37 °C. Table 1 provides the corrosion potential (E_corr_), corrosion current density (i_corr_), passivation potential (E_pass_), passive current density (i_pass_), the slope of the anodic curve (β_a_), the slope of the cathodic curve (β_c_), polarization resistance (R_p_), and corrosion rate obtained from potentiodynamic polarization tests for CP-Ti, Ti–5Cr, and Ti–5Cr–25Nb. The E_corr_ and i_corr_ of the three specimens were measured using the Tafel extrapolation method.

Ti–5Cr–25Nb demonstrates the highest E_corr_ value (–0.26 V), slightly exceeding CP-Ti (–0.30 V) and Ti–5Cr (–0.27 V). Moreover, Ti–5Cr–25Nb exhibits a higher R_p_ value compared to Ti–5Cr, along with a lower corrosion rate. This improvement is attributed to the enhanced corrosion resistance resulting from the addition of Nb to the alloy [20]. Additionally, all three specimens exhibit i_corr_ values in the order of 10^−^^9^ A/cm^2^, indicating excellent corrosion resistance. However, it is important to note that this does not necessarily imply comparable corrosion resistance, as the properties of the passivation layer on the alloy surface must also be taken into account. Regarding passivation capability, both E_pass_ − E_corr_ values for Ti–5Cr and Ti–5Cr–25Nb are lower than CP-Ti, suggesting that the addition of Cr and Nb accelerates the development of a passivation layer on the surfaces of Ti–5Cr and Ti–5Cr–25Nb. Pitting corrosion occurs in CP-Ti when the potential exceeds 1.2 V, whereas Ti–5Cr and Ti–5Cr–25Nb show no pitting corrosion at higher potentials, indicating the high stability of their passivation layers. This aligns with findings in literature, highlighting the ability of adding Cr and Nb elements to improve the stability of the passivation layer in Ti alloys [11,29]. Further analysis of the stability of the surface passivation layer for CP-Ti, Ti–5Cr, and Ti–5Cr–25Nb can be conducted through EIS testing.

#### 3.4.2. EIS Tests

**Figure 11:**
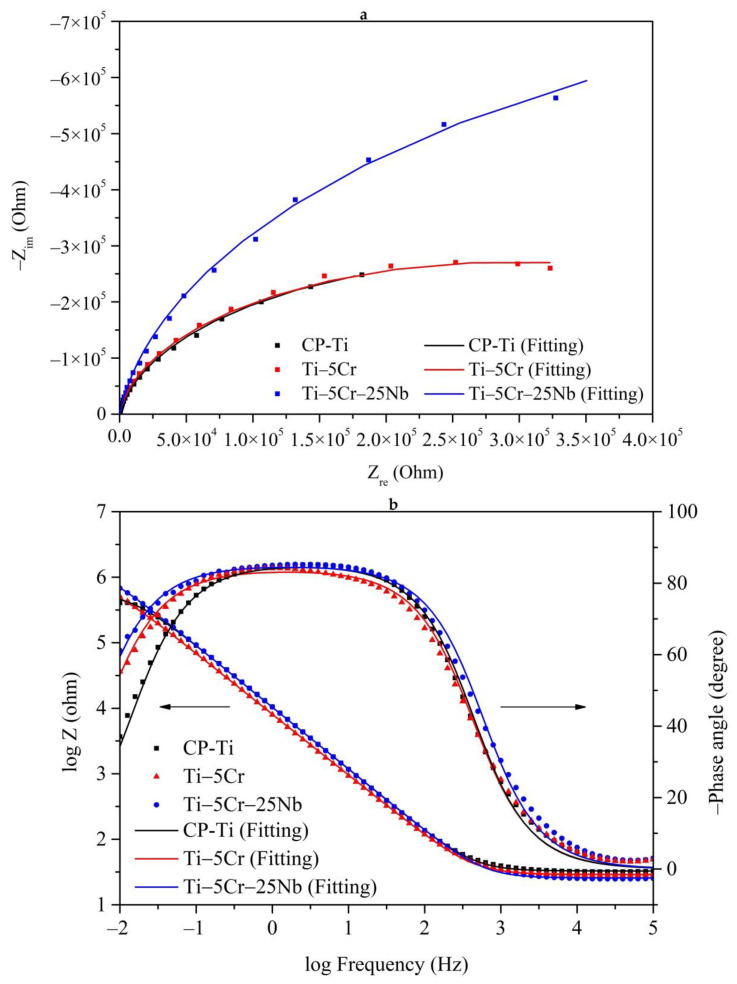
The EIS results for CP-Ti, Ti–5Cr, and Ti–5Cr–25Nb in a PBS solution at 37 °C with solid lines representing results simulated by Metrohm Autolab NOVA 2.1. (**a**) Nyquist plot and (**b**) Bode plot.

Figure 11 presents the EIS results for CP-Ti, Ti–5Cr, and Ti–5Cr–25Nb in a PBS solution at 37 °C, with solid lines representing results simulated by Metrohm Autolab NOVA 2.1. From the Nyquist plot (Figure 11a), it is evident that Ti–5Cr–25Nb exhibits the largest capacitance semicircle diameter, indicating optimal stability of its passivation film. In the Bode plot (Figure 11b), the slopes of the curves for CP-Ti, Ti–5Cr, and Ti–5Cr–25Nb in the high-frequency range (10^4^ to 10^5^ Hz) are close to 0, suggesting negligible experimental errors caused by electrolyte resistance [30]. The Bode-phase slopes for each specimen in the low-frequency range (10^−^^1^ to 10^1^ Hz) are close to 0, indicating the formation of a passivation film on the specimen surfaces. Furthermore, the negative phase angles of the Bode-phase curves for all specimens exceed 80°, indicating excellent capacitive behavior of the passivation film [31]. Additionally, Ti–5Cr–25Nb exhibits the highest impedance value at the termination frequency (10^−^^2^ Hz), indicating superior stability of its passivation film.

The EIS data for each specimen, including R_s_, CPE_1_, R_1_, deviation parameter for CPE_1_ (n_1_), effective constant capacitance value (C_eff_), and chi-square value (χ^2^), fitted based on the EEC in Figure 2 using Metrohm Autolab NOVA 2.1 software, are listed in Table 2. The formula for calculating C_eff_ is as follows [30]: $C_{eff,1}={{CPE}_{1}}^{\frac{1}{n_{1}}}{R_{1}}^{\frac{1-n_{1}}{n_{1}}}$. The χ^2^ values for each specimen were in the order of 10^−^^4^, indicating the validity of the equivalent circuit fitting results. Compared to CP-Ti, both Ti–5Cr and Ti–5Cr–25Nb exhibit low CPE_1_ values, suggesting more uniform passivation film structures with fewer defects [32]. Moreover, Ti–5Cr–25Nb possesses the highest R_1_ value, indicating the superior resistance to pitting corrosion of its passivation layer, attributed to the ability of Nb element to enhance the stability of the Ti alloy passivation layer. Overall, the combined results of potentiodynamic polarization and EIS demonstrate the outstanding corrosion resistance of Ti–5Cr–25Nb.

## 4. Conclusions

The XRD analysis revealed a predominant α′ + β phase in Ti–5Cr and Ti–5Cr–1Nb alloys, with the α′ phase diminishing as Nb content increases, ultimately leading to the exclusive presence of a meta-stable β phase beyond 5 wt.% Nb. This underscores the influence of Nb content on phase composition. Notably, the inhibition of ω phase formation in Ti–5Cr–xNb alloys (x = 20–40) significantly enhances deformability while reducing the elastic modulus, suggesting potential applications in flexible devices. Moreover, the addition of Nb elements enhances the corrosion resistance of Ti–5Cr alloys by stabilizing the surface passivation layer, as evidenced by the low corrosion rate (3.88 × 10^−^^5^ mm/year) observed in Ti–5Cr–25Nb alloy. Among the alloys studied, Ti–5Cr–25Nb demonstrated outstanding mechanical and corrosion properties, positioning it as a promising candidate for biomedical implant materials. Looking ahead, future research may focus on optimizing Nb content and alloy composition to tailor properties for specific applications, as well as exploring microstructural evolution and surface modification techniques to enhance biocompatibility. Overall, this study offers valuable insights into advanced Ti–Cr–Nb alloy design, suggesting avenues for further research and innovation in biomedical and engineering applications.

## Figures and Tables

**Table 1:** Corrosion characteristics obtained from potentiodynamic polarization tests for CP-Ti, Ti–5Cr, and Ti–5Cr–25Nb, including corrosion potential (E_corr_), corrosion current density (i_corr_), passivation potential (E_pass_), passive current density (i_pass_), anodic curve slope (β_a_), cathodic curve slope (β_c_), polarization resistance (R_p_), and corrosion rate.

Alloys	E_corr_ (V)	i_corr_ (A/cm^2^)	E_pass_ (V)	E_pass_ − E_corr_ (V)	i_pass_ (A/cm^2^)	β_a_ (V/dec)	β_c_ (V/dec)	R_p_ (MΩ·cm^2^)	Corrosion Rate (mm/year)
CP-Ti	−0.30	3.82 × 10^−^^9^	0.27	0.57	2.12 × 10^−^^6^	0.13	0.09	6.05	3.60 × 10^−^^5^
Ti–5Cr	−0.27	7.10 × 10^−^^9^	0.21	0.48	2.90 × 10^−^^6^	0.09	0.10	2.90	7.37 × 10^−^^5^
Ti–5Cr–25Nb	−0.26	4.03 × 10^−^^9^	0.27	0.53	2.23 × 10^−^^6^	0.11	0.08	4.99	3.88 × 10^−^^5^

**Table 2:** EIS data for CP-Ti, Ti–5Cr, and Ti–5Cr–25Nb, featuring electrolyte resistance (R_s_), constant phase element of the passivation layer (CPE_1_), polarization resistance of the passivation layer (R_1_), deviation parameter for CPE_1_ (n_1_), effective constant capacitance value (C_eff_), and chi-square value (χ^2^). Data were fitted based on the equivalent electrical circuit (EEC) model in Figure 2 using Metrohm Autolab NOVA 2.1 software.

Alloys	R_s_ (Ω·cm^2^)	CPE_1_ (10^−5^ F·cm^2^)	R_1_ (MΩ·cm^2^)	n_1_	C_eff_ (10^−5^ F·cm^2^)	x^2^ (10^−4^)
CP-Ti	30.72	3.54	0.60	0.93	4.47 × 10^−5^	1.19
Ti–5Cr	32.80	1.69	0.61	0.95	1.96 × 10^−5^	1.01
Ti–5Cr–25Nb	25.60	1.70	1.60	0.94	2.10 × 10^−5^	1.11

## Data Availability

Data are contained within the article.
